# Application of Exogenous GnRH in Food Animal Production

**DOI:** 10.3390/ani13121891

**Published:** 2023-06-06

**Authors:** A. H. M. Musleh Uddin, Kiro R. Petrovski, Yunmei Song, Sanjay Garg, Roy N. Kirkwood

**Affiliations:** 1 School of Animal & Veterinary Sciences, University of Adelaide, Roseworthy Campus, Roseworthy, SA 5371, Australia; roy.kirkwood@adelaide.edu.au; 2 Davies Livestock Research Centre, School of Animal & Veterinary Sciences, University of Adelaide, Roseworthy Campus, Roseworthy, SA 5371, Australia; kiro.petrovski@adelaide.edu.au; 3 Australian Centre for Antimicrobial Resistance Ecology, School of Animal & Veterinary Sciences, University of Adelaide, Roseworthy Campus, Roseworthy, SA 5371, Australia; 4 Clinical and Health Sciences, University of South Australia, Adelaide, SA 5000, Australia; may.song@unisa.edu.au (Y.S.); sanjay.garg@unisa.edu.au (S.G.)

**Keywords:** GnRH agonists, artificial insemination, reproduction, food animals

## Abstract

**Simple Summary:**

Optimum fertility requires the deposition of sperm at the appropriate time relative to ovulation, which can be achieved by using gonadotrophin-releasing hormone (GnRH) to control the time of ovulation. Following insemination, the establishment and maintenance of pregnancy and embryo survival are promoted by progesterone released from the ovarian corpora lutea, also under the control of GnRH-stimulated gonadotrophin release. This review examines the effect of the application of GnRH on the productivity and fertility of food-producing animals.

**Abstract:**

Over several decades, exogenous GnRH and agonists have been employed for controlling reproductive cascades in animals, and treating some reproductive morbidities. The administration of GnRH is used in animals to counter ovarian dysfunction, induce ovulation, and to increase conception and pregnancy rates. GnRH and its agonists are used in the treatment of cystic ovarian degeneration and repeat breeder syndrome. The development of protocols for GnRH administration by intramuscular injection, intramuscular or subcutaneous implants, and intravaginal deposition has empowered their clinical use worldwide. Currently, exogenous GnRH products are a central part of several pre- and post-breeding programs for the enhancement of fertility, including the control of estrous cycles and timing of ovulation, development of fixed-time artificial insemination protocols, improved embryo survival, and the treatment of reproductive morbidity. The aim of the present review is to summarize the application of exogenous GnRH agonists in food animal production.

## 1. Introduction

Reproductive performance is an important driver of economic and sustainable farming [1]; for cattle, it influences the efficiency of regular calving, optimum milk yield, and meat production. It is also dependent on the presence of suitable plans and strategies including several factors such as costs, the efficiency of utilization of farm facilities, environmental considerations, animal welfare, and public health. Poor reproductive performance is also a major reason for involuntary culling.

**Figure 1:**
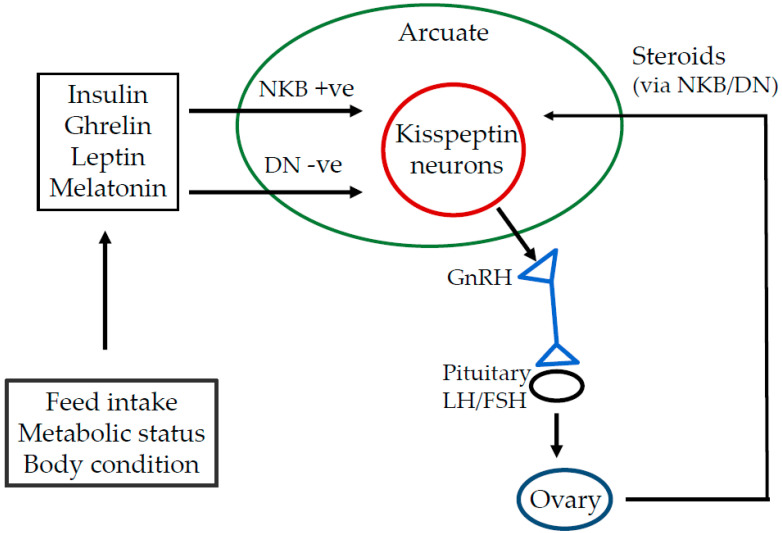
Involvement of neurokinin B (NKB), dynorphin (DN), and kisspeptin in the control of GnRH release.

Gonadotrophin releasing hormone (GnRH) plays a pivotal role in hormonal dynamics and reproductive efficiency in food animals. It is a peptide hormone synthesized and released from hypothalamic neurons under kisspeptin stimulation. Kisspeptin is released in response to neurokinin B (NKB) and is inhibited by dynorphin (DN), each being regulated by positive or negative hormonal signals including nutritional influences on metabolic status as indicated by, for example, responses in melatonin, leptin, ghrelin, and insulin (Figure 1). The GnRH enters the portal bloodstream at the hypophyseal region. Then, GnRH is transported to gonadotroph cells in the anterior pituitary gland where it triggers the GnRH receptor, and, ultimately, the synthesis and secretion of follicular stimulating hormone (FSH) and luteinizing hormone (LH) [2].

Currently, there are many identified naturally occurring GnRH agonists across vertebrate species [3]. Among them, GnRH I and GnRH II are the two forms of GnRH found in mammals, including pigs and cattle. GnRH I is the classical form of GnRH and is responsible for the regulation of LH and FSH secretion, being released in a pulsatile manner that regulates the secretion of gonadotropins from the pituitary gland. The functions of GnRH II have been reviewed previously [4], with reported involvement in ovarian steroidogenesis [5], progesterone synthesis [6,7,8,9], the development of follicles [10] and of the corpora lutea [5]. Interestingly, the GnRH1 receptor (GNRHR) and GNRHR2 show only 40% homology. Most mammalian species are not capable of generating GNRHR2 due to deletions and errors in gene coding. Only pigs among the livestock species retain the pertinent sequence code for GNRHR2 [11].

Being central to reproductive endocrinology, GnRH has the potential to be used in the management of reproductive performance in a variety of animal species and humans. For this reason, there is an ongoing interest in the use of exogenous GnRH for improving the reproductive performance of food-producing animals [12], with studies performed in different species, production systems, and climatic conditions. In this review, we will discuss the application of exogenous GnRH, or agonists, in food animal production, particularly regarding cattle and pigs.

## 2. Application of Exogenous GnRH in Controlled Breeding

### 2.1. Follicular Development and Ovulation Induction

Large doses of exogenous GnRH (100–500 µg) have been administered for the purpose of stimulating acyclic cows, and have shown greater efficacy in dairy cows [13] than in beef cows [12]. Interestingly, Ball [14] reported that these protocols had no effect on the calving to conception interval in dairy cows, while Beckett and Lean [15] found that the administration of GnRH before day 40 post-partum may reduce the time to first estrus but had no relation to the final reproductive performance of dairy cows.

In pigs, follicle growth from about 5 mm to ovulatory size (10 mm) primarily requires LH [16]. Due to the relatively low and batch-dependent LH-like activity, the estrus response to eCG injection has been noted to be dose-dependent. Injected doses of 363 to 600 IU resulted in 25% to 52% gilt estrus rates [17], while the injection of 725 to 1000 IU resulted in 70% to 100% estrus rates [18]. Similar to eCG, optimal GnRH dosages for inducing an ovulatory LH surge are product-dependent, being 10 µg for buserelin [19], 50 µg for gonavet [20], 10 µg fertilan [20], 25 µg for lecirelin [21], and 100–200 µg for intravaginal triptorelin [22,23,24,25,26,27]. Additionally, the efficacy of GnRH for ovulation induction is influenced by follicle size with sows having smaller follicles (<6.5 mm; [28]; <5.0 mm; [29]) at the time of treatment having poorer ovulation responses, presumably reflecting follicular immaturity.

### 2.2. Timed Artificial Insemination (TAI)

Timed artificial insemination (TAI) is an insemination timed relative to estrus detection with an assumed or controlled subsequent timing of ovulation, and it is commonly used in animal production [30]. In cyclic females, TAI protocols were established to control luteal and subsequent follicular dynamics to control estrus onset with subsequent satisfactory pregnancy rates. Protocols vary with species, reflecting species-specific follicular dynamics, corpora luteal sensitivities, and treatment logistics. The various TAI protocols involve the administration of exogenous gonadotrophins (equine chorionic gonadotrophin [eCG] and/or human chorionic gonadotrophin [hCG]) to drive ovarian follicular growth, GnRH to drive endogenous LH release, exogenous progesterone/progestin to suppress/synchronize estrus, prostaglandin F_2α_ (PGF) for luteolysis, or combinations of these hormones.

The artificial insemination of cattle after estrus detection resulted in a higher pregnancy per AI (P/AI) compared with an insemination after an ovulation synchronization protocol based on GnRH and PGF alone [31,32]. The stage of the estrous cycle [33,34] and the cyclic status [35] at the time of GnRH administration have also been shown to affect results [36]. Interestingly, due to prolonged luteal function with consequent impaired uterine involution and pyometra, an early resumption of postpartum ovarian cyclicity has been associated with reductions in the fertility of some dairy cows. In attempts to prevent these problems, Padula et al. [37] determined the potential for down-regulating pituitary gonadotrophin release using a deslorelin ear implant, and successfully delayed the resumption of postpartum ovarian cyclicity in recently calved Holstein cows.

In buffalo, GnRH was effective in inducing ovulation in around 80% of cows, making it suitable for use in timed AI [38,39,40,41]. The size of the ovarian follicle was important in timed artificial insemination protocols, as it affected the size of the subsequent corpus luteum, with larger corpora lutea having greater progesterone secretion. This, in turn, increased the chances of maintaining pregnancy and improving fertility in buffalo and cattle [42,43,44,45]. However, while cows have larger ovarian follicle diameters compared to heifers, this did not always result in improved reproduction, as in the older individuals hepatic steroid clearance could affect the result [46,47].

Buffalo pose a challenge for artificial insemination due to weak estrus signs and delayed ovulation [48]. However, there are still opportunities to overcome this challenge by utilizing timed artificial insemination during a specific ovulation window using protocols such as Ovsynch or a progesterone-based approach [49]. Research indicates that ovulation in beef and buffalo cows occurs between 65 and 75 h after CIDR removal, and that knowing this window is crucial for achieving optimal results [50,51]. However, administering GnRH/TAI at 72 h after a 7-day CIDR Co-synch may result in inadequate oocyte development when smaller follicles are subjected to ovulation, which could negatively affect embryonic and fetal survival because oocytes from small follicles have lower developmental competence compared to those from larger follicles [52]. In contrast, buffalo heifers receiving GnRH/TAI at either 72 or 84 h after CIDR removal showed good estrus intensity in 7-day CIDR Co-synch [48]. This is significant because estrus intensity positively affected conception rates in conventional farming systems [53].

The fertility of sows subjected to TAI has been variable, possibly due to variability in the wean-to-estrus interval affecting the synchrony of ovulation with insemination. Indeed, the interval between sperm deposition and ovulation is known to impact sow fertility [54]. It was demonstrated that the 24 h before ovulation was the optimum time for insemination, although the optimum window was also dependent on the age of the inseminated sperm [55]. A single dose of pLH, GnRH, or hCG administered at estrus detection can synchronize ovulation and allow a single insemination with acceptable fertility; there was no difference between treatment and control sows in terms of pregnancy rate and subsequent litter size [56]. Bai et al. [57] allocated primiparous sows to 1000 IU eCG at weaning followed by GnRH at estrus detection or 96 h after weaning, and also had a group of untreated controls. Treated sows were inseminated twice, about 8 and 32 h after GnRH, and control sows were inseminated at estrus detection and 24 h later. More sows receiving eCG were bred. The farrowing rates of bred sows were not different for eCG sows receiving GnRH at estrus detection and controls, but were lower for sows receiving GnRH at 96 h. No effects on subsequent litter sizes were evident [57]. These results lend support to the suggestion that, in sows, insemination without evidence of estrus is more likely to depress sow reproductive performance, with estrus expression being indicative of the presence of larger pre-ovulatory follicles.

Exogenous GnRH agonists in non-injection form, such as intravaginal implants of goserelin acetate or deslorelin [58,59] or a gel containing triptorelin acetate [22,28,60,61,62,63,64], have been used to control ovulation and so improve the management of artificial insemination in swine. Knox et al. [65] used mature gilts following estrus synchronization using the orally active progestogen, altrenogest, to perform a dose–response study with the vaginal deposition of different doses of triptorelin gel administered at different times after the last altrenogest feeding. They noted that the estrus response did not vary among treated gilts but that ovulation intervals were shorter in GnRH treatment groups, and showed the highest ovulation responses when deposited at 120 h after the last altrenogest feeding.

### 2.3. Fixed Timed Artificial Insemination (FTAI)

**Figure 2:**
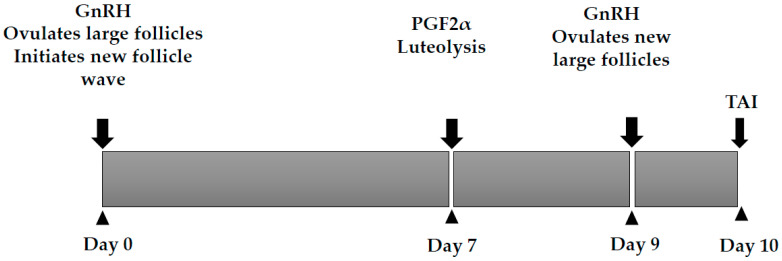
Ovsych protocol for TAI in cattle.

Fixed timed artificial insemination (FTAI) protocols based on exogenous GnRH are commonly used in cows [66,67,68,69], and, to a lesser extent, pigs [56,70,71]. The protocols consist of GnRH administration to initiate LH release and the ovulation of the follicle, and, in sows, insemination without estrus detection, while, in cattle, initiation of renewed follicular dynamics 1–2 days later. After an initial GnRH injection in dairy cattle, PGF2α was administered at 7 days to induce luteolysis, followed by a second dose of GnRH given to provide ovulation synchronization [72,73]; this protocol is known as Ovsynch (Figure 2). A variation, Co-Synch, is usually used in beef cattle because the cows are inseminated at the time of the second GnRH to minimize animal handling [74]. The first GnRH was responsible for a 44–54% ovulation rate in dairy cows [75,76], 60% in beef cows, and 56% in beef heifers [34]. If the first GnRH failed to synchronize a follicular wave, the ovulation following the second GnRH may be poorly synchronized [77], resulting in unexpectedly low pregnancy rates. To increase the number of ovulated cows, pre-synchronization with one or two doses of PGF followed by the first GnRH dose was introduced [78,79]. The efficacy of the pre-synchronization is yet to be firmly established.

FTAI protocols in sows and gilts are used to control ovulation and allow insemination without the detection of estrus while maintaining their fertility. Martinat-Botté et al. [23] treated altrenogest-synchronized gilts with an injection of buserelin at different times following the last altrengest feeding, and sows at different times after weaning. They noted an improved synchronization of ovulation in gilts treated at 104 h compared to 120 h, and in sows treated at 94 h compared to 104 h. Clearly, the timing of treatment relative to degree of follicular maturation will influence the response to GnRH administration. Non-injection GnRH treatment using triptorelin in gel has also been used extensively in sows [80,81,82]. This involves vaginal deposition 96 h after weaning, with insemination 24 h later, and the reproductive performance has been commercially acceptable. Of interest, however, is that while farrowing rates were generally comparable, in sows not exhibiting standing estrus at the time of insemination the farrowing rate was very low [83,84]. The lack of estrous behavior presumably reflected inadequate follicular development resulting in non-ovulation or the ovulation of poor quality oocytes, and thus poor farrowing rates.

### 2.4. Embryo Survival and Pregnancy Rate

Embryo mortality is an important reproductive constraint in farm animals, resulting in pregnancy loss or reduced litter sizes, depending on the number of embryos originally present. Cattle first-service embryo mortality rates ranged from 10% to 40% and were up to 65% for repeat breeders [85]. Injecting GnRH at the time of artificial insemination may increase the pregnancy rate in cows. However, effects have been equivocal due to inadequate animal numbers and differences in experimental design. It is noteworthy that Peters [12] reported that a meta-analysis indicated improved pregnancy rates in GnRH-treated cows.

A recent comparison was made between two GnRH products, the agonist dephereline and natural GnRH, administered in cows following the removal of a 5-day progesterone-containing CIDR treatment [86]. Thereafter, FTAI was performed in cows that were heat-stressed, or not, at the time of insemination. Cows treated with dephereline showed greater ovulation and pregnancy rates compared to those receiving the natural GnRH, especially under conditions of heat stress. Although speculative, these findings may suggest the heat stress inhibition of hypothalamic/pituitary function with a reduced GnRH release adversely affecting reproductive function, and that exogenous GnRH can override this. In another stress situation, excitable Nellore cows had higher blood cortisol levels [87], and a clear link between stress and cortisol levels has been established [88]. In excitable Nellore cows, it has further been observed that the administration of GnRH at 7 days after TAI improved pregnancy rates at 30 days but not gestational losses assessed at 60 days [87]. This effect was presumably due to GnRH-induced LH release providing early luteal support. In contrast, an evaluation of the impact of GnRH treatment in dairy cows experiencing heat stress during AI, and 5 days after AI, noted no improvement in pregnancy rates, gestational loss, or P4 concentrations [89].

It has been noted that the injection of GnRH into sows at artificial insemination increased the numbers of embryos in sows underfed in their previous lactation, and in primiparous sows, and this was associated with increased blood progesterone concentrations [90]. The mechanism underpinning this effect was not determined, although it has been previously documented that a lower magnitude LH surge was associated with subsequently lower blood progesterone concentrations, with the suggestion that a smaller surge will lead to poorer follicular luteinization [91]. Although speculative, it is possible that a GnRH injection at mating could enhance an otherwise attenuated LH surge, improve luteal quality, and promote improved pregnancy maintenance.

It is known that GnRH injections in the mid-late luteal phase in non-gravid cows will elicit increased progesterone secretion [92]. The increased progesterone secretion also stimulates the production of estradiol-17β, with Mann et al. [93] concluding that the GnRH attenuated or weakened the luteolytic signal allowing embryos more time to develop their own luteotrophic capability.

GnRH was examined as a protector of pregnancy in buffalo cows, related to a suboptimally functioning corpora lutea. Its administration in the various stages of pregnancy, usually after day 25, improved final pregnancy rates [94,95], probably by decreasing early embryonic loss [96]. The role of GnRH in improving pregnancy rates is an ongoing area of research and is of particular interest in some physiologic states, such as in dairy cows that suffer from heat stress-induced early embryonic losses. In sheep, it was reported that GnRH administered to ewes 12 days post-mating resulted in improved embryo survival [97]. However, in another study, the administration of GnRH and progesterone 4 and 12 days post-breeding improved embryo survival but did not increase the pregnancy rate in ewes [98]. Unfortunately, there is a dearth of data concerning the influence of exogenous GnRH for improving embryo survival in sheep and goats.

Several studies have investigated the effects of the LH agonist, hCG, in reducing embryo mortality in pigs. Injecting 750 IU on day 12 of the estrous cycle resulted in prolonged luteal survival and increased progesterone production [99,100]. Additionally, exogenous hCG improved luteal viability, as well as angiogenesis in endometrial and luteal tissues. It also had a positive effect on the PGE2–PGFM ratio during the estrous cycle, which decreased luteal cell apoptosis during early pregnancy in sows and gilts [99]. It remains to be determined whether GnRH, via the stimulation of endogenous LH, can substitute hCG, although, presumably, it will require a more sustained activity than what is currently available.

Several researchers have reported that a single injection of GnRH between 11 and 13 days after insemination resulted in an elevation of progesterone and an improved pregnancy rate of about 10% in cows [101,102,103,104]. However, others found no relationship between exogenous GnRH administration and pregnancy performance [105]. The lack of a GnRH effect on pregnancy rates does not necessarily mean a lack of efficacy. The result of any study is a comparison between a treatment group and a control group, but in the event that the control group fairs well, the treatment ‘does not work’. This is well illustrated in a study on the effects of GnRH injected into buffalo cows on day 35 after insemination [95]. Cows with a blood serum concentration of pregnancy-associated glycoproteins (PAGs) of less than 2.5 ng/mL were considered at a higher risk of embryo mortality. Only within this subpopulation, treatment with GnRH significantly increased PAGs between the 28th and 60th days of pregnancy, and markedly reduced embryo/pregnancy loss.

For cows, the placentation process begins around days 28–32 of pregnancy and concludes between 40 and 45 days [106,107]. The elevation in PAG levels indicates the expansion of trophoblastic tissue as its quantity is linked to the growth of the placenta [108,109]. The trophoblastic cells play a crucial role in prostaglandin metabolism and they can convert luteolytic PGF2α into luteotrophic PGE2, which ensures the maintenance of the corpus luteum and adequate levels of progesterone necessary for the survival of the conceptus [110,111]. When GnRH was administered on the 5th or 15th day [112], or on the 12th day [113], there was no noticeable improvement in pregnancy rates. Conversely, administering GnRH via subcutaneous implantation on the 27th day following insemination significantly reduced embryonic loss between 45 and 90 days in cows that had developed accessory corpora lutea [96].

The establishment of pregnancy in buffaloes requires a gradual increase in progesterone secretion during the first 2–3 weeks following mating. To support pregnancy, various treatments prolonging the lifespan of the corpus luteum have been employed, including GnRH treatment on the 5th or 25th day after insemination [114,115]. The GnRH treatment on the 5th day after AI increased progesterone secretion and enhanced the chances of a successful pregnancy in buffaloes bred during periods of increasing photoperiod, while treatment on the 25th day resulted in a higher pregnancy rate [114,115].

### 2.5. Treatment of Reproductive Morbidity

The use of GnRH as a treatment for some reproductive morbidities in food animals is not well established [116,117], but is well recognized for others, such as cystic ovarian degeneration in cattle [118,119,120]. Early studies found that serial administrations of different doses of GnRH were unable to initiate ovulation in anestrous cows [121,122]. Repeat breeding syndrome, i.e., a requirement for repeated inseminations without detectable abnormalities, is a major reproductive problem in dairy and beef cows, and increases the costs of production. The use of GnRH agonists to modify the condition of repeat breeding cows has been investigated [117]. Various studies reported that GnRH agonists administered coincident with insemination had positive effects on conception and pregnancy rates, finding a 10% to 18% more successful pregnancy in repeat breeder cows compared to untreated groups [12,117,123]. Further, a buserelin dose–response effect on the conception and pregnancy rates of repeat breeder cows has been identified, with 20 µg resulting in better pregnancy rates than 10 µg [117]. Similarly, the injection of 2.5 times the label dose (250 µg) of dephereline at 5 to 7 days after insemination increased pregnancy rates in repeat breeder cows more than 100 µg did [124], with both doses resulting in accessory corpora lutea. Taken together, these data suggest that repeat breeding may be a consequence of inadequate GnRH stimulation, and that this is a compensable effect that can be addressed by administering higher than label exogenous doses.

The inclusion of eCG in a controlled ovulation protocol prolonged stimulation during a follicle’s dominance, and estradiol secretion elevated the concentrations of follicular cytochrome enzyme P450 17α hydroxylase [125,126]. This increase induced the LH surge, leading to ovulation in cattle [125]. Therefore, combining eCG on day 5 with GnRH on day 9 of a FTAI protocol can enhance the induced LH release effect, resulting in the ovulation of the dominant follicle in a shorter time in suckled beef cows. This also resulted in a significantly larger corpus luteum on day 9 after ovulation, evidenced by a higher concentration of serum progesterone on day 12. Similar findings were reported by other researchers, who observed greater luteal volume and progesterone concentration in anestrous cows that received eCG two days before intra-vaginal device removal in an 8-day FTAI protocol, at 10 and 15 days after ovulation [127]. These results align with the concept that the progesterone concentration plateau occurs between 10 and 14 days after ovulation [128], and that luteal size is closely related to luteal function. The measurement of luteal area had the strongest association with luteal function during its development [129]. Furthermore, cows having higher progesterone concentrations sooner after ovulation are generally associated with greater fertility [130].

Following a progesterone-based protocol for estrus induction, the reproductive performance of anestrous ewes receiving treatment with nanoencapsulated GnRH was evaluated as a potential alternative to eCG [131]. The authors developed a nano-GnRH formulation with distinct physicochemical features that yielded favorable outcomes on ovulation and luteal function in various animal species, including goats, sheep, and rabbits [132,133]. Their results illustrated that GnRH administration, as a component of ovulation control protocols, resulted in a significant increase in conception, lambing, and fecundity rates with a tendency towards larger litter sizes compared to those treated with eCG. However, various studies have indicated potential adverse impacts of eCG on embryo survival, particularly during the pre-implantation stage, when utilizing either in vivo [134] or in vitro [135,136] techniques. For instance, in Chios-breed ewes, the use of eCG for superovulation, and, to a lesser extent, for estrus synchronization, can impact glycosidase activity in the genital tract, potentially leading to adverse effects on embryo survival and pregnancy maintenance before the maternal recognition of pregnancy [134]. As a substitute for eCG, nanoencapsulated GnRH can be employed in progesterone (CIDR)-based estrus induction regimens for sheep [131]. 

The use of GnRH agonists in the treatment of cystic ovarian degeneration is established in cows [118,119,120,137], and there is an indication that double injection has an effect on this morbidity in sows [138]. In fact, in cows, GnRH is the treatment of choice for the undifferentiated cystic ovarian degeneration, independent of the type (i.e., follicular or luteal cyst/s). Responsiveness to the first administration of GnRH has been suggested to be an indicator for prognosis, with cows not responding to the first treatment having a lower likelihood of return to fertility [137].

## 3. Future Research

The application of GnRH for controlling reproductive performance in food production animals has shown great promise. Consequently, there is a growing interest in utilizing exogenous GnRH to enhance the reproductive performance of food production animals. To explore the potential use of GnRH in the veterinary field in more detail, further studies should be conducted across a range of animal species, production systems, and climatic conditions. Such research would provide invaluable insights into GnRH, ultimately facilitating its optimal use in maximizing its performance in the practical aspects of food animal production.

## 4. Conclusions

Exogenous GnRH and agonists have been extensively researched as a promising strategy for regulating and managing fertility in female animals, offering promising approaches for controlling and enhancing reproductive performance. GnRH and agonists in FTAI have been demonstrated as an efficient and successful reproductive protocol that can be incorporated into farming systems, due to an improved understanding of the endocrine system that governs follicular development and ovulation. FTAI protocols result in a decrease in both the number of inseminations and seminal doses required for achieving pregnancy, along with satisfactory reproductive performance. Injectable forms of GnRH have been developed and used in animals to achieve maximum outcomes, but there is limited information about non-injectable forms of GnRH and agonists that are more animal welfare friendly. Furthermore, the efficacy of non-injectable GnRH in controlling ovarian function, inducing ovulation, and maintaining pregnancy in food animals has received limited attention.
